# Continuous cough monitoring: a novel digital biomarker for TB diagnosis and treatment response monitoring

**DOI:** 10.5588/ijtld.22.0511

**Published:** 2023-03-01

**Authors:** S. Huddart, L. Asege, D. Jaganath, M. Golla, H. Dang, L. Lovelina, B. Derendinger, A. Andama, D. J. Christopher, N. V. Nhung, G. Theron, C. M. Denkinger, P. Nahid, A. Cattamanchi, C. Yu

**Affiliations:** 1 UCSF Center for Tuberculosis, University of California San Francisco, San Francisco, CA, USA; 2 Division of Pulmonary and Critical Care Medicine, San Francisco General Hospital, University of California San Francisco, San Francisco, CA, USA; 3 Infectious Diseases Research Collaboration, Kampala, Uganda; 4 De La Salle Medical and Health Sciences Institute, Center for Tuberculosis Research, City of Dasmariñas, Cavite, The Philippines; 5 Hanoi Lung Hospital, Hanoi, Vietnam; 6 Department of Pulmonary Medicine, Christian Medical College, Vellore, India; 7 DSI-NRF Centre of Excellence for Biomedical Tuberculosis Research, and SAMRC Centre for Tuberculosis Research, Division of Molecular Biology and Human Genetics, Faculty of Medicine and Health Sciences, Stellenbosch University, Tygerberg, Cape Town, South Africa; 8 Department of Medicine, Makerere University College of Health Sciences, Kampala, Uganda; 9 Vietnam National Tuberculosis Control Program, Hanoi, Vietnam; 10 Division of Infectious Diseases and Tropical Medicine, Center of Infectious Diseases, Heidelberg University, Heidelberg, Germany; 11 German Center for Infection Research (DZIF), Heidelberg University Hospital Partner Site, Heidelberg, Germany

Dear Editor, Cough is a key symptom of TB and many other respiratory conditions. Until recently, assessments of cough burden have been largely subjective and limited to self-report. Quantitative data on the timing and frequency of coughing could help to identify patterns of cough that are unique to respiratory diseases, facilitating diagnosis and monitoring of response to treatment. Such data can now be obtained through smartphone applications which support longitudinal continuous cough monitoring as patients follow their normal daily routines. To further explore its potential, we conducted 14 days of smartphone-based longitudinal continuous cough monitoring of people with presumptive TB at health centers in five countries (Uganda, South Africa, the Philippines, Vietnam and India) using the Hyfe Research application,^1^ and compared patterns of cough between people with microbiologically confirmed TB, clinical TB, and other respiratory diseases (ORD).

Eligible participants were 18 years or older and had new or worsening cough for at least 2 weeks. Participants were excluded if they had taken TB medicines in the previous 12 months or taken medication with anti-mycobacterial activity in the past 2 weeks. Participants were provided with smartphones loaded with the Hyfe Research app to continuously monitor participant cough for 14 days after enrollment. Participants recorded for a median of 23.9 hours a day.^2^ TB status was classified based on positive sputum Xpert® MTB/RIF Ultra (Cepheid, Sunnyvale, CA, USA) or mycobacterial culture results (microbiologically confirmed TB) or empiric TB treatment initiation (clinical TB). Empiric TB treatment decisions were made by non-study clinicians based on medical history (TB symptoms and risk factors), physical examination, and/or chest X-ray findings. Data and code are made available at https://github.com/skhuddart/TBcoughmonitoring.

As of August 15, 2022, a total of 565 participants completed cough monitoring and had results of TB reference standard testing. The median age was 38 years (interquartile range [IQR] 26–51), 54.5% of participants were female, and the median BMI was 22.1 kg/m^2^ (IQR 19.0–26.0). In the past 7 days, 21.4% of participants had smoked; 13.3% of participants were living with diabetes and 12.7% were living with HIV. The median self-reported duration of new or worse cough before the care-seeking appointment was 28 days (IQR 15–58). Based on sputum Xpert and culture results, 144 (25.5%) participants had microbiologically confirmed TB, 48 (8.5%) were treated for clinical TB and 373 (66.0%) were classified as having ORD. Among the 144 participants with microbiologically confirmed TB, 93.8% (135/144) reported initiating TB treatment. TB treatment was initiated a median of 1 day (IQR 0–2.5) after study enrollment. On the first recording day, the overall median cough count per hour (medCPH) was 5.0 (IQR 3.0–9.0) (Figure). On Day 1, participants with microbiologically confirmed TB had a significantly higher medCPH (8.0, IQR 3.5–19.0) than participants with ORD (5.0, IQR 3.0–8.0; *P* < 0.001). In contrast, there was no significant difference in medCPH between participants with clinical TB (5.0, IQR 2.0–9.0 vs. ORD; *P* = 0.76). By Day 14 of recording, the overall medCPH had fallen to 3.5 (IQR 2.0–6.0) and had decreased significantly compared to Day 1 values in all three groups (all *P-*values < 0.001). In addition, at Day 14 there was no significant difference in medCPH between participants with ORD (3.0, IQR 2.0–6.0) vs. either microbiologically confirmed TB (4.0, IQR 2.0–8.0; *P* = 0.38) or clinical TB (3.0, IQR 2.0–4.9; *P* = 0.24). Participants with TB showed unique cough frequency trajectories early in treatment that were distinct from those of participants with ORD. Furthermore, cough frequency improved more rapidly among participants with microbiologically confirmed TB than other groups. These findings support quantitative cough monitoring as a potential treatment monitoring tool.^3^

Further work is needed to assess the correlation between cough frequency and established treatment response biomarkers (such as time to positivity in liquid culture). In addition, future studies should explore whether cough features, including cough frequency, are able to improve the accuracy of current TB screening algorithms. The use of cough-based biomarkers for TB screening may help reduce the overtreatment of people without TB and improve detection of TB when missed by routine symptom-based screening procedures. In summary, continuous cough monitoring should be further explored as a non-invasive biomarker for TB diagnosis and treatment monitoring.

**Figure:**
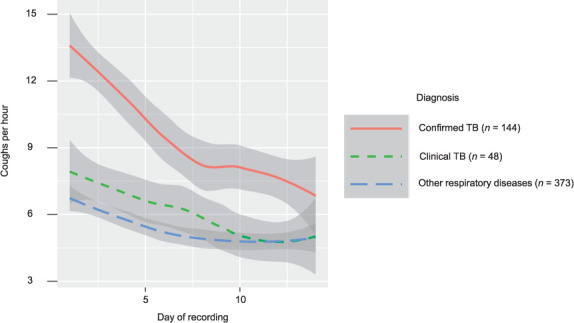
Cough frequency trajectories by diagnosis. Local regression (LOESS) smoothing is applied to medCPH values by day within each diagnosis group. Shaded grey regions indicate LOESS confidence intervals. medCPH = median cough count per hour; LOESS = LOcally Estimated Scatterplot Smoothing.
